# The bipolar two-syndrome concept: questioning the shaping of a circular argument for subtyping a dimensional disorder

**DOI:** 10.1186/s40345-022-00278-0

**Published:** 2022-12-18

**Authors:** Gin S. Malhi, Erica Bell

**Affiliations:** 1 Academic Department of Psychiatry, Northern Clinical School, Faculty of Medicine and Health, Kolling Institute, The University of Sydney, Sydney, NSW Australia; 2 Northern Sydney Local Health District, CADE Clinic and Mood-T, Royal North Shore Hospital, Level 3, Main Hospital Building, Sydney, NSW 2065 Australia; 3 Department of Psychiatry, University of Oxford, Oxford, United Kingdom

In a recent paper published in this journal by Tondo and colleagues (Tondo et.al. 2022), the authors set out to compare the characteristics of bipolar disorder patients diagnosed as bipolar I and II according to DSM-5. The paper examines more than 1300 subjects and predictably identifies differences of severity across a range of parameters. Somewhat surprisingly, based on this, the article concludes that *“bipolar II is dissimilar to, but not necessarily less severe than bipolar I. The several prominent dissimilarities between bipolar I and bipolar II support the hypothesis that they represent distinct syndromes in need of individualised treatments”*. Unfortunately, the conclusion does not accord with the findings, and although we argue against two ‘kinds’ of bipolar disorder—there are indeed two problems for us to consider.

First, the study is fundamentally flawed because of the way the samples are generated for comparison. Second, the interpretation of the *“various differences”* is subject to a form of ‘interpretation bias’. We therefore consider each of these in turn and offer an alternative interpretation that accords with our longstanding position namely, the bipolar construct is dimensional and that the creation of bipolar II on arbitrary grounds remains a wholly theoretical construct that lacks any meaningful foundation and clinical utility.

## The fundamental flaw

The study (Tondo et.al. 2022) examined 1377 patients attending a specialist mood disorders clinic. They were diagnosed as having either bipolar I or bipolar II disorder, based on DSM-5 criteria. The criteria for bipolar disorder in DSM-5 distinguish bipolar I and II disorder based on the severity of manic symptoms, the degree of impairment these cause, and whether the person has experienced psychosis. The latter confers a diagnosis of bipolar I, as does hospitalization.

However, it is important to note that other than psychosis, none of the clinical symptoms are unique to either supposed subtype. Further, the use of hospitalisation as a criterion for diagnosis has been widely criticised and is recognised as being somewhat absurd; at best, it serves as a proxy of severity. In other words, other than psychosis there are no manic symptoms that distinguish either subtype and clinically, the differentiation is made purely based on severity and duration of symptoms. This manifestly speaks to a dimensional model.

Indeed, a dimensional model is precisely what we have argued for (Malhi 2021) and suggested that perhaps the lower limit of four days should be reduced further to capture the additional presentations that are alluded to by DSM-5 with the inclusion of diagnostic ‘categories’ (e.g., depressive episodes with short-duration hypomania) within its less well-known sections such as “conditions for further study” (American Psychiatric Association 2022). Predictably, if severity has been used to separate symptoms in patients and subsequently assign diagnostic categories, then the resulting diagnoses will necessarily (and self-evidently) differ in this regard. This is to be expected. Clearly, a fundamental error has occurred involving circular reasoning, and the purported findings of this study are not surprising as they  simply derive from the straightforward  bisection of each dimension.

## Interpretation bias

The authors claim they found dissimilarities between bipolar II and bipolar I, and that these are consistent with the two being “distinct syndromes”. The dissimilarities span descriptors such as employment, morbidity, specific item scores on clinical scales, and the treatment that patients had received. The authors themselves summarised these as bipolar II cases having “*higher socioeconomic and functional status*” and “*more prominent and longer depressions*”. However, nearly all the findings in putative bipolar II patients regarding the description of the disorders and associated morbidity are in keeping with depression simply being less severe, specifically without psychomotor symptoms and those reflective of psychosis (e.g., paranoia), and a greater weighting towards depression as compared to manic symptoms. Thus, predictably, the treatments for so-called bipolar II patients are also skewed towards antidepressant use and away from the use of antipsychotics and lithium (Malhi 2020).

We are puzzled as to how any of these differing grades of severity can be construed as reflecting specificity, let alone supporting the concept of a distinctive syndrome. In fact, we would have been more surprised if the findings had been anything other than those reported—given the a priori separation of patients into bipolar I and bipolar II according to DSM-5 criteria. Thus, the authors have not only made an initial error in design in relation to how they have defined their samples for comparison, but they have then subsequently accentuated this problem by interpreting their findings through a subjective lens.

## Alternative interpretation

**Fig. 1:**
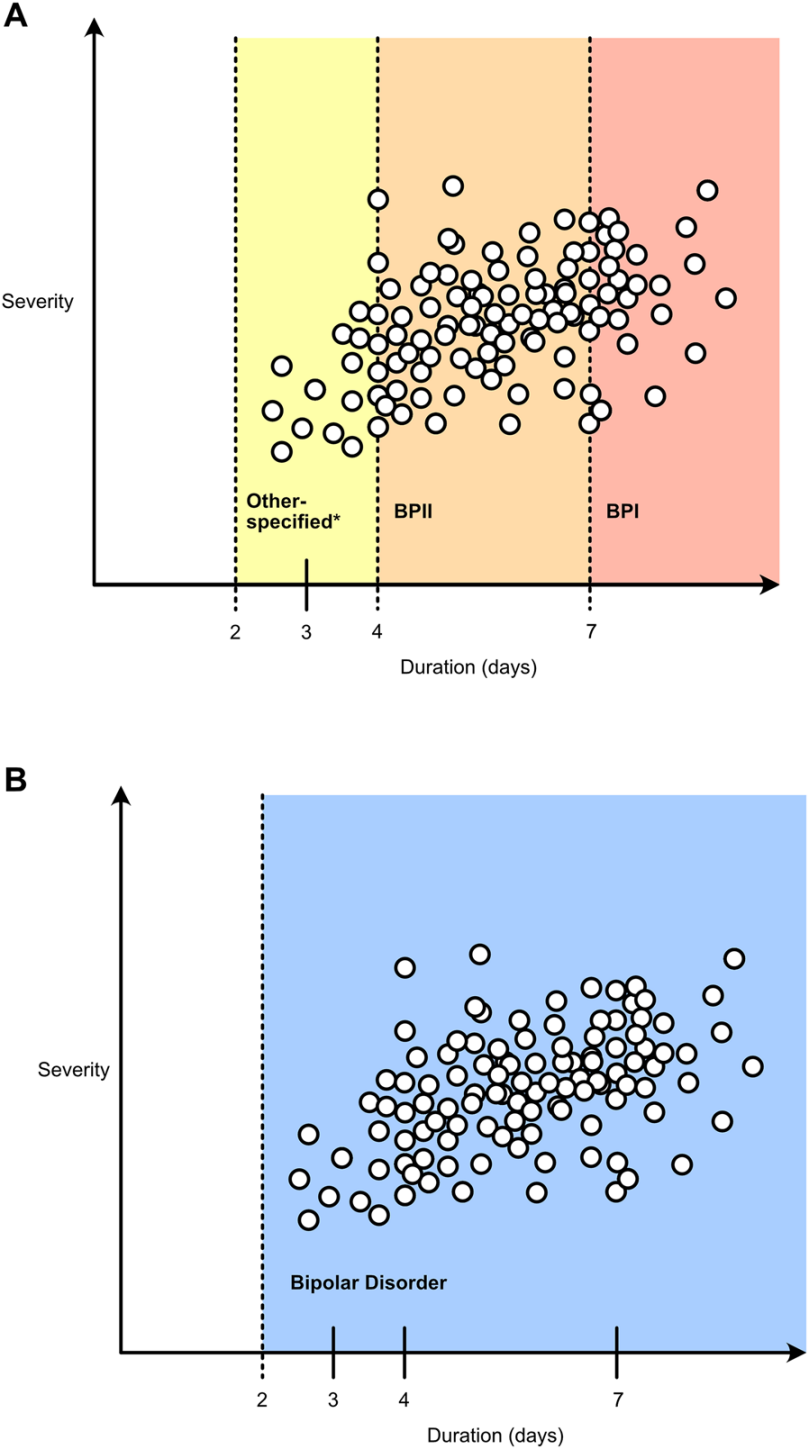
Schematic illustrating approaches to classifying bipolar disorder. The *top panel* illustrates the perspective taken by Tondo and colleagues, wherein they acknowledge that a dimensional perspective reflects clinical reality, but within this entity, subcategories have been created and labelled as bipolar I and II. The problem with this approach is that the boundaries of these subtypes are unclear and difficult to define. The *bottom panel* depicts our model in which bipolar disorder is a single entity comprising symptoms that vary according to severity and duration. In addition, the lower boundary of bipolar disorder in terms of duration of symptoms is a minimum of 2 days, and once the threshold of a manic episode has been reached, then the syndrome can be specified by simply indicating the number of days of manic symptoms the patient has experienced. In this way, treatment can be tailored to the individual’s symptoms and severity and impact of these symptoms. *Other-specified refers to the ‘Short-duration hypomanic episodes (2–3 days) and major depressive episodes’ diagnosis listed within the ‘Other-specified and Related Disorders’ category in DSM-5

The introduction of bipolar II as a clinical concept was a logical and pragmatic step informed by the limited information available at the time. Originally bipolar II was developed as a practical ‘model’, which would undergo refinement in light of emergent evidence. This approach is in keeping with the framework for diagnoses proposed by Robins and Guze (Robins and Guze 1970). But no new insights have come to light and the concept has not been refined. Instead, it has been reified in the absence of any substantive corroborative research. Remarkably, after more than four decades of research, there is no unequivocal evidence to support the division of bipolar disorder along the lines of bipolar I and bipolar II. In fact, research has time and again produced results similar to the current study—suggesting that the disorder should not be dichotomised (See Fig. 1).

Perhaps even more surprising is the fact that the solution is staring us in the face. The logical interpretation of research findings that accords with the clinical reality that we see, is that “there is no category of bipolar II”. And that when this term is used in clinical practice it merely captures, in a somewhat haphazard manner, milder forms of bipolar disorder that are invariably harder to differentiate from comorbid illnesses such as personality disorder and anxiety. In addition, these diagnoses are more easily obscured by substance misuse, and hence form a diagnostic cloud that surrounds bipolar II diagnosis in clinical practice. Indeed, we appreciate the difficulty in identifying and managing the full spectrum of bipolar presentations, and we therefore recommend readers consult the recently published guidelines for the management of mood disorders (Malhi et.al. 2021), which include treatment advice for all manifestations of bipolar disorder. The fact of the matter is that the evidence is clear and straightforwardly interpretable. The difficulty is that the solution seems to be unpalatable, and it is not completely clear why this is so. Possible reasons include the concern that too much has been invested in subtyping the disorder and that over nearly half a century, too many people have already been assigned a diagnosis of bipolar II.

Nevertheless, we continue to champion a dimensional perspective, in which the duration criteria could be lowered, and the number of days patients experience manic symptoms could simply be specified rather than arbitrarily drawing artificial lines at 4 days and 7 days. We suggest that perhaps future research should invest in returning to not having a priori assumptions as regards categories, and instead examine clinical data along with biological and psychological data sets using modern methods of establishing linkages such as network analyses (that have been applied to symptoms) (Bryant et.al. 2017). Further, it may be possible to harness the benefits of analyses drawing on artificial intelligence to interrogate data to determine new means of drawing connections that have clinical salience; however, this does require relinquishing our dependence on the current taxonomies.

An alternative hybrid model remains in which there are semblances of categories reflecting underlying variance at subordinate levels, but because these overlap considerably at the phenomenological level, they blur into a continuum that is difficult to dissect using clinical parameters alone. This possibility needs to be borne in mind but should not determine clinical practice as yet, until such purported subgroupings can be identified, and even then, they only carry import if they confer clinical advantage. And so, while we respect the attempt to address the question using research, we remain unconvinced by the findings of this particular study, which to our minds supports our dimensional perspective and negates the concept of bipolar II further.

## Data Availability

Not applicable.
